# Regenerative Medicine in the Management of Musculoskeletal Pain: Mechanisms, Clinical Applications, and Future Directions

**DOI:** 10.7759/cureus.116523

**Published:** 2026-09-19

**Authors:** Salah N El-Tallawy, Joseph V Pergolizzi, Tariq A Alzahrani, Giustino Varrassi, Abdullah T Alsubaie, Robert B Raffa, Mamdouh M Haddara, Rania S Ahmed, Radwa H Ahmed, Aalaa M Doheim, Mohamed S Nagiub

**Affiliations:** 1 Anesthesia and Pain Management, King Khalid and King Abdulaziz University Hospitals, College of Medicine, King Saud University, Riyadh, SAU; 2 Anesthesia and Pain Management, Faculty of Medicine, Minia University, Minya, EGY; 3 Anesthesia and Pain Management, National Cancer Institute (NCI), Cairo University, Cairo, EGY; 4 Pain Management, NEMA Research Inc., Naples, USA; 5 Anesthesia and Pain Management, King Khalid University Hospital, College of Medicine, King Saud University, Riyadh, SAU; 6 Pain Medicine, Fondazione Paolo Procacci, Rome, ITA; 7 Anesthesia and Pain Management, King Abdulaziz University Hospital, King Saud University Medical City, Riyadh, SAU; 8 School of Pharmacy, Temple University, Philadelphia, USA; 9 Pain Medicine, King Faisal Specialist Hospital and Research Centre, Riyadh, SAU; 10 Medicine, College of Medicine, Alfaisal University, Riyadh, SAU; 11 Clinical Pathology Department, Faculty of Medicine, Assiut University, Assiut, EGY; 12 Anesthesia and Pain Management, Assiut University Hospitals, Assiut, EGY; 13 Medicine, College of Medicine, Badr University, Badr City, EGY

**Keywords:** functional improvement, gene therapy, musculoskeletal pain, osteoarthritis, pain relief, platelet-rich plasma, regenerative medicine, stem cells and exosomes, tendinopathy and muscle injuries, tissue engineering

## Abstract

Musculoskeletal disorders represent a leading cause of global disability. Common causes of musculoskeletal pain include anomalies, repetitive strain, sports injuries, obesity, aging, inflammation, and degeneration. These conditions manifest as osteoarthritis, tendinopathy, low back pain, erosions, and fractures. Management of musculoskeletal pain uses multidisciplinary, multimodal approaches, including pharmacologic, non-pharmacologic, physical therapy, and surgical interventions. Conventional treatments such as simple analgesics, nonsteroidal anti-inflammatory drugs, corticosteroids, opioids in selected cases, and even surgery may provide symptomatic control at the expense of adverse effects such as gastrointestinal bleeding, addiction, dependence, and cartilage erosion.

Regenerative medicine has emerged as a promising therapeutic approach for musculoskeletal pain encompassing modalities such as platelet-rich plasma (PRP), mesenchymal stem cells (MSCs) and their derivatives, gene therapy, and tissue-engineered scaffolds. These interventions may alleviate musculoskeletal pain through multiple biological mechanisms, including modulation of inflammation, desensitization of nociceptors, enhancement of tissue repair, and stimulation of cellular regeneration.

A targeted literature search was conducted between 2011 and 2026 across major databases, including PubMed, MEDLINE, Web of Science, and Embase. The inclusion criteria focused on high-quality publications addressing the role of regenerative medicine modalities in musculoskeletal pain. The exclusion criteria included non-musculoskeletal pain, animal studies, and other publications such as editorials and case reports.

This review shows promise and supports the potential role for regenerative therapies in the management of musculoskeletal conditions, but their effectiveness, safety, and feasibility vary substantially by modality, indication, product standardization, and study quality. The study identifies important knowledge gaps, limitations, and future directions in regenerative medicine, while also underscoring the need for better standardization and stronger comparative evidence.

## Introduction and background

Musculoskeletal pain is a major global health burden that affects people all around the world regardless of age and gender. This challenging condition has a substantial impact on the quality of life [1]. Acute musculoskeletal complaints affect approximately 47% of the general population [2], while chronic presentations can be seen in 20-33% of individuals, translating to an estimated 1.75 billion people globally [2,3]. Chronic musculoskeletal pain is a leading cause of disability, contributing significantly to years lived with disability [1,3]. In addition, the economic burden of musculoskeletal disorders exceeds USD $1 trillion annually, due to the compounding effects of healthcare expenditures, lost productivity, and long-term disability pensions [2,3]. Various bone injuries and anomalies impair the natural healing process, highlighting the critical clinical necessity for advanced strategies for bone regeneration [4].

Current management of musculoskeletal pain is typically multimodal, combining non-pharmacological interventions (such as physical therapy), pharmacological strategies (such as simple analgesics, nonsteroidal anti-inflammatory drugs (NSAIDs), and adjuvants), and surgical or interventional procedures in selected cases [3]. However, each of these approaches has significant limitations.

From a pharmacologic standpoint, NSAIDs provide symptomatic relief for many musculoskeletal conditions, such as osteoarthritis, low back pain, and acute musculoskeletal pain. The pharmacokinetic profile of NSAIDs carries risks of gastrointestinal toxicity, cardiovascular events, and renal dysfunction, particularly with chronic use [5]. Opioid therapy has limited efficacy for chronic musculoskeletal pain and carries substantial risks of addiction, dependence, tolerance, and overdose [6]. Corticosteroid injections provide temporary benefit, but evidence suggests possible cartilage degeneration with repeated administration [7].

Surgical intervention also has limitations. Although some comparative evidence suggests that many procedures yield outcomes similar to those of conservative therapy or sham surgery [3], overutilization of surgical interventions persists. Procedures such as knee arthroscopy for degenerative joint disease and shoulder arthroscopic decompression for impingement are frequently performed despite research showing they offer little to no long-term benefit over conservative therapy or sham interventions [8]. Given the potential complications and the limited ability of surgery to restore normal tissue function and mobility, new and effective therapeutic approaches are clearly needed [9].

Conventional musculoskeletal interventions are fundamentally palliative; they are designed to alleviate pain without addressing the underlying biological processes of tissue degeneration and impaired healing. The pathophysiology of musculoskeletal disorders involves sustained pro-inflammatory cytokine cascades, reduced cellular regenerative capacity, and progressive tissue loss [10,11].

Regenerative medicine aims to address these degenerative mechanisms. The National Institutes of Health (NIH) defines it as “the process of creating living, functional tissues to repair or replace damaged cells, tissues, or organs” [12]. Parallel to this, the FDA defines regenerative medicine as “a general approach to restore, replace, or recreate cells, tissues, or organs to treat or mitigate disease [13]. Within this field, a critical biological distinction must be maintained between regeneration and repair. Regeneration refers to the precise restoration of native tissue architecture, such as hyaline cartilage rich in type II collagen. In contrast, repair denotes fibroplasia and scar formation, typically yielding a biomechanically inferior fibrocartilage rich in type I collagen, and durable human histological proof of true cartilage regeneration in osteoarthritis remains limited [14,15].

As an interdisciplinary field, regenerative medicine aims to restore or re-establish normal biological function that has been compromised due to aging, disease, trauma, or congenital defects. Unlike conventional analgesics that temporarily suppress nociception, regenerative medicine targets the degenerative microenvironment. By reducing local inflammation and limiting progressive tissue loss, these modalities shift the clinical objectives from symptom control toward structural repair. By integrating tissue engineering, cellular therapies, gene therapy, and biomaterials, regenerative strategies stimulate healing by enhancing endogenous repair mechanisms and facilitating the regeneration of damaged tissues [12,16-19].

Given the rapid expansion of regenerative therapies across musculoskeletal medicine, clinicians are increasingly faced with a complex and evolving therapeutic landscape that spans established interventions, emerging biologics, and investigational regenerative technologies. Rather than attempting an exhaustive systematic evaluation of all available studies, this review provides a comprehensive summary of contemporary regenerative medicine approaches developed for musculoskeletal pain and details their underlying biological mechanisms. Additionally, the manuscript focuses on comparative clinical effectiveness and safety profiles of these modalities, while addressing ethical considerations, regulatory challenges, and current limitations. It provides a balanced overview of regenerative strategies currently influencing musculoskeletal pain management, while highlighting areas of promise, ongoing uncertainty, and existing limitations. It also outlines future clinical direction as well as research trajectories necessary to advance the field.

## Review

Methods

This narrative review is designed to provide a clinically oriented review of regenerative medicine approaches for musculoskeletal pain. It focuses on the underlying mechanisms, representative clinical findings, and safety considerations using a narrative rather than a systematic approach.

A targeted literature search was conducted using PubMed, MEDLINE, Web of Science, and Embase to identify relevant studies published during the last 15 years, between January 2011 and January 2026. The search strategy used combinations of keywords, including musculoskeletal pain, osteoarthritis, tendinopathy, low back pain, myofascial pain, regenerative medicine, platelet-rich plasma, stem cells, mesenchymal stem cells, exosomes, tissue engineering, and gene therapy. Two independent reviewers screened the titles and abstracts to determine eligibility.

The inclusion criteria prioritized high-quality evidence, including systematic reviews, meta-analyses, randomized controlled trials (RCTs), major observational studies, and clinical practice guidelines. Additional sources were identified through manual screening of reference lists of key articles. A range of regenerative medicine strategies were considered, including platelet-rich plasma (PRP), stem cell therapy, exosomes, gene therapy, tissue engineering, and combination therapies.

Exclusion criteria comprised non-English publications and articles focused on non-musculoskeletal pain (e.g., cancer pain, postoperative pain, and primary neuropathic pain). We also excluded editorials, conference abstracts, letters, case reports, and expert opinions. A total of 4,620 publications were identified through a literature search covering the past 15 years. Two independent reviewers screened the selected articles using the same eligibility criteria. After removing duplicate and non-English publications, irrelevant studies, articles evaluating non-musculoskeletal pain, and other records that did not meet the inclusion criteria, 46 articles were ultimately included in the final review. Steps of the identification of studies, screening, and selection via databases and registers are illustrated in Figure 1.

**Figure 1:**
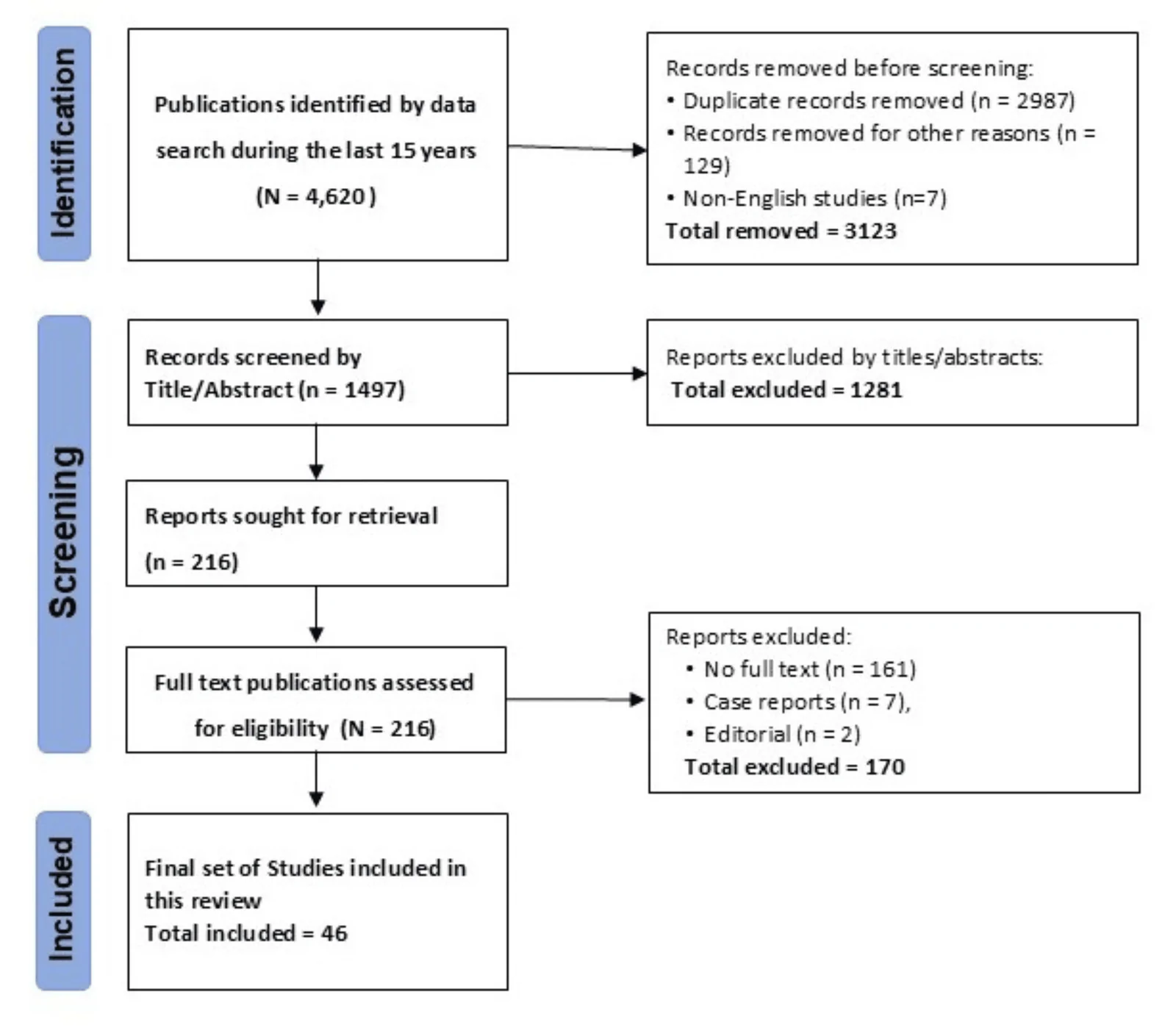
Identification of studies via databases and registers.

Study selection was consistent with narrative review methodology, focusing on scientific quality and clinical significance rather than a predefined systematic screening protocol. Accordingly, articles were selected based on relevance to clinical applications, mechanisms of action, safety, and outcomes related to pain and functional improvement, with the primary goal of providing a clinically meaningful synthesis of the available literature rather than an exhaustive systematic analysis.

Given the narrative nature of this review, formal risk-of-bias assessment and quantitative synthesis were not performed. Instead, findings were synthesized descriptively to provide a clinically meaningful overview of current evidence and emerging trends.

No ethical approval was required as this narrative review is based on previously published articles and does not contain any new studies conducted on human participants or animals.

Regenerative medicine modalities in the management of musculoskeletal pain

Platelet-Rich Plasma (PRP)

PRP is an autologous biological therapy prepared from the patient’s own blood, thereby minimizing the risk of immunogenic reactions or disease transmission [20,21]. It is typically prepared using a two-step centrifugation process to separate blood components based on their density. During the first centrifugation, erythrocytes settle to the bottom of the suspension. A leukocyte-rich buffy coat forms above this layer [21,22]. The remaining plasma layer, which contains concentrated platelets, is then collected and subjected to a second centrifugation to further isolate and pellet the platelets [21,23,24]. This process yields a significantly higher platelet concentration than that found in whole blood. The platelet content of PRP is enriched to three to nine times the baseline platelet concentration (e.g., 6 × 1011 platelets/ml) [25,26].

PRP injections modulate inflammation and promote tissue repair; therefore, they are used for several indications, including osteoarthritis, tendinopathy, and low back pain. However, clinical results remain variable, particularly in muscle injuries, where RCTs have shown inconsistent benefits [21,27,28].

The therapeutic rationale for PRP is based on the activation-dependent release of multiple growth factors, including platelet-derived growth factor (PDGF), transforming growth factor-beta (TGF-β), insulin-like growth factor (IGF), fibroblast growth factor (FGF), and vascular endothelial growth factor (VEGF). These factors support tissue repair by promoting healing, regeneration, and functional recovery through targeted growth factor release [21,26,27].

Proposed mechanisms involve the stimulation of angiogenesis, enhancement of cellular migration and proliferation, and the promotion of extracellular matrix deposition. At the same time, a dense leukocyte-rich or highly concentrated pro-inflammatory profile can promote myoblast proliferation, which may increase the likelihood of scar formation, fibrosis, and heterotopic ossification in muscle tissues. The duration of clinical benefits varies across studies, although pain reduction is generally sustained for six to 12 months following injection [26,28].

Evidence supporting PRP varies by indication and formulation. In adhesive capsulitis, a recent systematic review and meta-analysis pooling 11 studies with 971 patients compared intra-articular PRP injections with corticosteroids for adhesive capsulitis. The analysis found that PRP provided superior long-term efficacy in terms of improved pain, shoulder function, and range of motion [29]. In contrast, clinical evidence regarding PRP on muscle injuries shows no consistent benefit over rehabilitation alone in time to return to play (RTP) or reinjury rates (level 1b-2b) [30-32]. Building on these condition-specific findings, emerging data revealed that optimal treatment outcomes depend on important factors, including PRP concentration and early intervention. Regarding the frequency of injections, studies revealed that two injections are more effective than one injection [29]. Furthermore, PRP demonstrates potential synergistic benefits when used alongside other treatments [33].

Recent data from the guidelines of the 2025 update of the American Society of Interventional Pain Physicians (ASIPP) revealed that intradiscal injections of PRP and mesenchymal stem cells (MSCs) in the lumbar region are supported by level III evidence, as are lumbar epidural PRP injections, whereas PRP injections for lumbar facet joints and sacroiliac joints are supported by level IV evidence [34]. PRP injections for various musculoskeletal indications are expanding rapidly. While individual centrifugation systems have cleared the FDA 510 (k) pathway for creating autologous plasma formulations, the therapeutic clinical indications themselves remain investigational and lack formal approval by the FDA. PRP injection is considered relatively safe because it includes autologous injections of the patient’s own tissues. PRP can be administered as a stand-alone intervention or used in combination with other procedures [35,36]. The reported adverse effects are usually minor and include allergic reactions, a risk of heterotopic ossification (HO), and potential pain exacerbation or delayed recovery driven by proinflammatory cytokines. Despite increasing clinical use, variability in PRP preparation protocols, including platelet concentration, leukocyte content, and activation methods, makes direct comparison across studies difficult and contributes to inconsistent findings [37].

Platelet-Poor Plasma (PPP)

PPP is the plasma fraction obtained after centrifugation of whole blood and contains fewer platelets compared to PRP, but it still contains bioactive proteins and cytokines [36,37]. It is increasingly viewed as part of regenerative medicine because it is an autologous biologic derived from the patient's blood and contains bioactive proteins that may support tissue repair [38]. However, the evidence base is less robust than for PRP, largely because it lacks comparable PDGF content [39].

PPP is typically prepared from anticoagulated whole blood. After initial processing, the PPP layer is separated and further centrifuged, after which the upper 3-6 mL of PPP is collected and injected into the target injured site [22,25,40]. Its relatively low platelet content may support myogenic differentiation, enhance muscle healing, and potentially lower the risk of reinjury. However, clinical evidence remains limited and inconclusive [32].

The major components of PPP are fibrinogen, fibronectin, thrombin, and other soluble materials. The biological effects of PPP are to participate in hemostasis and coagulation, to act as a cell attachment vector, and to promote mitosis of fibroblasts and epithelial cells [35]. It may also contain regulatory levels of cytokines such as IL-6, tumor necrosis factor-α (TNF-α), and VEGF, which help regulate inflammation and support tissue repair without driving persistent inflammation or fibrosis [35,41]. In addition, VEGF may promote neovascularization, improve perfusion of the injured muscle, and enhance the delivery of oxygen and nutrients needed for effective healing. By modulating inflammatory cytokine release, PPP can limit excessive collagen deposition, thereby reducing fibrotic scar formation and attenuating chronic inflammation that could impair muscle function [35,41].

PPP also facilitates the proliferation of muscle cells and key reparative cells, including fibroblasts, thereby contributing to tissue regeneration and functional recovery after injury [41,42].

PPP may be used for muscle injuries due to its ability to drive true myogenic differentiation while maintaining better control over the inflammatory response. It promotes recovery of the injured muscles by providing a more balanced approach with less tissue inflammation [32].

Injection of PPP into injured muscle tissue induces the immature myoblast cells toward the muscle differentiation pathway, rather than the proliferative pathway observed with PRP injection [32,43]. The reduced platelet content in PPP can also minimize the risk of a prolonged inflammatory reaction, which in turn could lead to chronic inflammation or persistent pain after muscle injuries [35,43].

When using autologous blood products, PPP is a good option if the therapeutic goal is to enhance myogenic differentiation while attenuating pro-inflammatory and pro-fibrotic signaling [44]. PPP may be favored for patients who might be sensitive to high platelet levels or who have previously experienced complications with PRP [32]. In acute muscle injury, PPP may help support faster recovery while avoiding excessive inflammation, making it a suitable option for myogenic regeneration. Clinical evidence on PPP for muscle injuries suggests RTP, but data are limited and non-definitive [32,45,46]. However, there is no strong evidence that PPP is superior to either PRP or structured rehabilitation programs alone in facilitating RTS [37]. Similar to PRP, PPP is an autologous tissue product; it often does not require additional regulatory approval for certain homologous applications; however, its efficacy still requires clinical evidence (Table 1) [32].

**Table 1:** Comparison of platelet-rich plasma (PRP) and platelet-poor plasma (PPP).

	PRP	PPP
Definition	Autologous plasma enriched with platelets, with variable leukocyte content [20,21,25].	Lower-platelet plasma fraction obtained after PRP preparation, with preserved soluble factors [35,36,37].
Preparations	Prepared from whole blood by centrifugation to concentrate platelets, with composition depending on the system used [20,25,26].	Collected from the upper plasma layer after PRP separation [22,35,40].
Mechanisms	Promotes repair and angiogenesis through growth-factor release, but may also drive inflammatory or fibrotic signaling [20,21,25].	Provides a gentler biologic signal that may support regeneration while limiting excess inflammation [32,35,44].
Indications	Used for osteoarthritis, tendinopathy, low back pain, and other soft-tissue disorders [29,30,34].	Mainly explored for acute muscle injury and myogenic regeneration [32,37,46].
Advantages	Best-studied blood-derived ortho-biologic and widely used in practice [29,34,37].	Potentially less inflammatory, with a theoretical anti-fibrotic benefit [35,41,44].
Limitations	Highly variable preparation methods and inconsistent results in muscle injury [30,37].	Evidence is limited, and clinical use is less standardized [37,46].
Safety	Usually well tolerated; musculoskeletal use is often off-label despite device clearance [34,37].	Regulatory support remains limited for specific indications [32,37].
Evidence	Strongest support is in osteoarthritis and selected tendinopathies, with weaker support in muscle injury [29,32,34].	Promising but still preliminary, especially for muscle recovery [32,37,44].

Mesenchymal Stem Cells and Their Derivatives in Musculoskeletal Regeneration

Traditional (intact) mesenchymal stem cells (MSCs): MSCs are spindle-shaped cells that adhere to plastic in culture and show multilineage differentiation potential that can differentiate into fibroblasts and other mesodermal cell types [22]. The International Society for Cellular Therapy (ISCT) characterizes these fibroblast-like adult multipotent progenitor cells as having the capacity for self-renewal and differentiation in vitro, particularly into several cell types, such as osteoblasts, adipocytes, chondrocytes, and tenocytes [30,46,47].

MSCs can be isolated from fetal and adult tissues. Fetal sources include umbilical cord materials like Wharton's jelly, which offers allogeneic cells with immunomodulatory effects, placental tissues, and laboratory-reprogrammed induced pluripotent stem cells (iPSCs) [26]. Adult tissue sources encompass bone marrow, adipose tissue, liver, skin, synovium, and dental pulp [48-50].

MSCs may support musculoskeletal regeneration through several complementary mechanisms. They can differentiate into chondrocytes and osteocytes, release paracrine growth factors that enhance endogenous repair pathways, and modulate immune responses by shifting macrophages toward anti-inflammatory phenotypes. Mechanistically, MSCs modulate TGF-β signaling, Wnt/β-catenin pathways, and SOX9-driven transcriptional activation with downstream COL2A1 and aggrecan expression, thereby promoting chondrogenesis and extracellular matrix synthesis [22,51,52].

Under biochemical or mechanical stimuli, MSCs also generate cartilage-specific extracellular matrix elements such as type II collagen and glycosaminoglycans, which support tissue integration and functional recovery in osteoarthritis [22,51,52]. Following administration of MSCs, they migrate to the injury site, regulate inflammatory responses, and differentiate into functional cells to repair or replace damaged tissues or organs [30,53]. Clinical benefits include pain relief and improved cartilage glycosaminoglycan (GAG) content; however, challenges remain, including hypertrophic potential and source variability [10,28,30,50].

In knee osteoarthritis, bone marrow-derived MSCs combined with hyaluronic acid (HA) showed sustained clinical improvement superior to HA alone for a four-year follow-up period [10,28,30]. Furthermore, these applications demonstrated evidence of cartilage regeneration on imaging; however, the available human evidence remains insufficient to confirm durable hyaline cartilage regeneration histologically in a reproducible manner [10,28,30,50,54,55]. In contrast, peripheral blood-derived MSCs with HA demonstrated considerable improvement in clinical and radiological scores for significant chondral defects over a two-year follow-up period [30,55]. Additionally, intradiscal MSC injections have shown promising preliminary results in reducing inflammation and promoting disc cell proliferation in patients with low back pain [46]. MSCs can also accelerate fracture healing and ligament repair in experimental and early clinical studies [30,46]. Treatment with MSCs showed sustained clinical improvements across several applications, with no clinically significant adverse effects in multiple studies [7,46,49,50].

MSCs have recently emerged as a potential treatment for systemic lupus erythematosus (SLE) due to their capacity to target T cells, exert anti-inflammatory effects, alleviate symptoms, and help restore immune balance [30,54]. They have also gained attention as a therapeutic modality for the treatment of rheumatoid arthritis (RA), based on their anti-inflammatory and immunoregulatory properties, paracrine signaling, and the ability to differentiate into various cell types [30,54,55].

In clinical practice, MSC therapy has generally shown a favorable short-term safety profile, with most adverse events being mild and local. Accordingly, clinical use should remain limited to properly regulated and ethically approved settings [30].

Adipose tissue-derived regenerative therapies: Adipose tissue has emerged as an attractive source for regenerative medicine. It contains adipose-derived mesenchymal stromal/stem cells (ASCs), pericytes, endothelial and immune cells, extracellular matrix (ECM), growth factors, and signaling molecules. The regenerative potential of adipose-derived products appears to depend more on paracrine signaling, immunomodulation, extracellular-vesicle release, and restoration of the local tissue microenvironment [56-58]. Microfragmented adipose tissue (MFAT) maintains important cell-matrix interactions and may provide a more physiologic regenerative microenvironment. MFAT therefore represents a potentially integrated therapeutic product combining regenerative cells, bioactive mediators, and a natural tissue scaffold [58-60]. Adipose-derived products can also modulate macrophage activity and inflammatory signaling, potentially shifting the local environment from persistent inflammation toward tissue repair [59,61,62].

RCTs have demonstrated that intra-articular MFAT has been associated with improvements in pain and function. A prospective randomized trial found sustained improvement with both MFAT and PRP over 24 months, without a consistent difference between treatments. Thus, current evidence supports MFAT as a promising orthobiologic option for knee osteoarthritis (OA) [57,63,64].

Adipose-derived therapies have also been investigated in focal cartilage lesions and tendinopathies. They produced better clinical outcomes in patients with focal chondral lesions of the knee. These findings support the regenerative potential of adipose-derived products in musculoskeletal tissue repair, although the evidence remains limited [65-67].

Adipose-derived approaches have further been explored in degenerative disc disease. However, these indications remain at an earlier stage [57,67]. Overall, adipose tissue should be viewed as a multicomponent regenerative platform rather than simply a source of stem cells. Current evidence is limited by differences in processing methods, variability between adipose-derived products, and the limited number of high-quality randomized trials [58-60].

Mesenchymal stem cell-derived extracellular vesicles (MSC-EVs): MSC-EVs are gaining attention in regenerative medicine for musculoskeletal system repair by integrating cell therapy, biomaterial, and immune engineering approaches [68]. These nanoscale, membrane-bound vesicles released by MSCs, encompassing exosomes, microvesicles, and apoptotic bodies, are natural secretory products of MSCs. MSC-EVs are emerging as potential solutions to the key limitations of intact cellular therapies. Extracellular vesicles (EVs) can be modulated by cellular preconditioning or vesicle engineering, and thus represent a versatile tool for regenerative medicine to improve the function of the targeted tissues [22,68]. Compared to conventional MSC therapy, EVs offer significant advantages, including low immunogenicity, superior penetration of biological barriers, and stable cryopreservation parameters [10,69].

The preparation of MSC-EVs typically involves several steps, starting with cell extraction from various tissue sources, followed by cell culture and expansion, differential ultracentrifugation or ultrafiltration for vesicle isolation, purification, and storage. Once ready, they are utilized as targeted drug delivery vesicles through exogenous cargo loading, administration, and targeted therapeutic action. However, their regulatory framework remains complex and varies across countries, rendering their standardization challenging [55,70].

The biological effects of MSCs are driven mainly by paracrine signaling. MSCs secrete growth factors such as FGF, hepatocyte growth factor (HGF), and VEGF that promote local cell proliferation. Concurrently, these cells modify the host immune response by suppressing T-cell activation and inducing a regulatory phenotype shift. These effects create a pro-regenerative tissue healing environment [30].

MSCs also release extracellular vesicles (~30-5000nm), a heterogeneous group of membrane-bound particles that include exosomes (~30-150 nm), microvesicles (~100-1000 nm), and apoptotic bodies (up to several micrometers). These cell-free vesicles play a major role in the therapeutic benefits observed with MSC therapy [10,30,46].

EVs inherently transport proteins, lipids, and nucleic acids and can also be bioengineered as targeted drug delivery systems [71,72]. Depending on both EV cargo and the target cell type, EVs can modulate key cellular processes such as proliferation, differentiation, senescence, and apoptosis, in addition to influencing immunomodulation, blood coagulation, and angiogenesis [73,74].

MSC-EVs serve as central regulators of musculoskeletal repair by modulating inflammation, matrix turnover, and intercellular signaling across muscle, bone, cartilage, tendon, and intervertebral disc tissues, and appear clinically safe so far, with only mild, predominantly local side effects reported [40,75]. EVs from adipose and immune cells also influence glucose metabolism and muscle function through pathways like peroxisome proliferator-activated receptor gamma (PPARγ), shaping both normal physiology and conditions like obesity and muscular dystrophy [68,76]. In bone, MSC-EVs help maintain metabolic homeostasis by supporting extracellular matrix remodeling and mineralization, enhancing osteoblast-mediated bone formation, stabilizing osteoclast activity, and thereby mitigating bone loss [77]. They further contribute to musculoskeletal balance through immune modulation, the promotion of angiogenesis, and support for bone innervation [77,78].

In osteoarthritis, MSC-EVs promote chondrocyte growth and migration, decrease apoptosis and aging, boost matrix production, shield cartilage from breakdown, and influence immune regulatory signaling [79]. Additionally, MSC-EVs promote tendon and ligament healing by modulating immunity, promoting angiogenesis, enhancing the proliferation and migration of tendon fibroblasts, and supporting extracellular matrix buildup [80]. Similarly, in intervertebral disc degeneration, EVs influence disease progression by altering inflammatory and apoptotic pathways, particularly through microRNA transport affecting macrophage polarization and ECM balance [81,82]. Overall, EVs serve as central messengers across the musculoskeletal system, mediating cellular crosstalk and promoting regenerative responses, processes that can be stimulated even by physiological triggers like exercise [83,84].

Safety records demonstrate an excellent safety profile in several clinical trials, with mostly minor side effects like local injection-site reactions. Serious side effects, such as infections or increased malignancy incidence, are very rare with aseptic technique [30,85,86]. However, the FDA has reported serious adverse events associated with unapproved, illegally marketed, or unregulated products of MSCs, including blindness following intra-retinal injection, tumor formation, and life-threatening infection [87].

The main challenges of using MSC products include high manufacturing costs ($5,000-20,000+), manufacturing complexity, and a lack of standardized protocols that limit accessibility. Furthermore, variations in clinical efficacy are partly attributable to cell processing differences and limited long-term durability data extending beyond two years (Table 2) [86,88].

**Table 2:** Comparative analysis of traditional mesenchymal stromal cells (MSCs) and MSC-derived extracellular vesicles (MSC-EVs). EVs: extracellular vesicles; miRNA: micro ribonucleic acid; mRNA: messenger ribonucleic acid.

	Traditional MSCs	MSC-derived EVs
Definition	Plastic-adherent multipotent stromal cells with self-renewal and differentiation potential [30,44].	Cell-free vesicles released by MSCs, including exosomes and related EV subtypes [10,22,80].
Preparations	Derived from bone marrow, adipose tissue, umbilical cord, and other tissues, followed by culture expansion [30,48].	Obtained from conditioned MSC media and then isolated, purified, and sometimes bioengineered [55,82].
Mechanisms	Act mainly through paracrine signaling and immunomodulation, with some contribution from endogenous repair support [22,30,41].	Transfer proteins, lipids, miRNA, and mRNA to regulate inflammation, angiogenesis, apoptosis, and matrix turnover [83,84,85].
Indications	Studied for osteoarthritis, cartilage repair, bone healing, disc disease, and inflammatory musculoskeletal disorders [30,46,54].	Investigated for muscle, bone, cartilage, tendon, ligament, and disc regeneration, and as delivery platforms [40,89,90-96].
Advantages	Broad biologic activity and more mature translational experience than EVs [30,46].	Cell-free, easier to store, less immunogenic, and more engineerable [10,22,80].
Limitations	High cost, manufacturing complexity, source variability, and heterogeneity [49,97,98].	Isolation and potency testing are not yet standardized, and regulatory classification is evolving [55,82,92,93,97,98].
Safety	Generally encouraging safety in controlled studies, but unregulated products remain a concern; use is mostly investigational [30,99].	Early safety is promising, but long-term safety and regulatory pathways remain uncertain [40,97].
Evidence	More established than EVs, though still limited by heterogeneity and translational barriers [30,46,49,50].	Highly promising, but clinical evidence is earlier and less standardized than for whole-cell MSCs [80,87,98,100].

Exosomes: Exosomes represent a specific class of small EVs that cells release as membrane-bound particles, typically measuring 30-150 nm in diameter [10,44]. MSC-derived exosomes carry a rich payload of proteins, bioactive lipids, microRNAs, and mRNA, facilitating precise intercellular signaling [46,85].

Exosomes can be generated by various functional cell types, meaning they exhibit significant heterogeneity rather than being a uniform component [89]. However, they are prone to rapid degradation and changes within hours of production, whether naturally secreted or artificially engineered. This characteristic has posed challenges for quality control testing and has led to the development of innovative technologies like lyophilization and cryopreservation to preserve their structure and biological activity [90].

Exosomes typically result from the fusion of multivesicular bodies (MVBs) with the plasma membrane, releasing intraluminal vesicles (ILVs) into the surrounding extracellular microenvironment, hence the term exosomes [91]. Their formation begins with early endosomes generated via endocytosis of extracellular components; as the membrane buds inward, it traps proteins, lipids, and other molecules to form the ILVs that accumulate inside the endosomes, maturing into MVBs [22,92]. The lack of regulatory standards, along with the diversity of manufacturing techniques, renders their standardization challenging. Accordingly, the FDA has alerted consumers that no exosome products are currently approved [93].

Recent clinical trials have compared the ability of MSC-derived EVs to replicate the actions of their parent cells and have concluded that MSC-EVs can deliver comparable benefits to MSCs in areas such as the repair of injury and tissue regeneration [94-96], anti-inflammatory activity [97], cell proliferation and migration [96,98], and the promotion of collagen synthesis [88,93] and angiogenesis [96,98,99]. These findings indicate that MSC-EVs could serve as an alternative to MSCs as a therapeutic tool, while eliminating some of the side effects associated with cell-based therapies [94,100].

Numerous studies on MSC-derived exosomes have demonstrated good safety profiles alongside encouraging preliminary results, including faster tissue healing and improved functional recovery [10,101]. For musculoskeletal conditions, they hold potential in osteoarthritis management, as well as tendon and ligament repair via diverse administration routes, including topical, intra-articular, and even inhalation approaches [102,103].

In osteoarthritis, MSC-EVs appear to act through TGF-β-related chondrogenic signaling, Wnt/β-catenin regulation, and SOX9-associated upregulation of COL2A1 and other matrix genes, thereby supporting chondrocyte phenotype maintenance, matrix preservation, and suppression of catabolic signaling [68,71,72,79,82].

Overall, exosomes represent a promising future direction in regenerative medicine by providing a more standardized treatment and engineerable cell-free therapeutic platform than whole-cell products. However, manufacturing, quality control, regulatory standardization, and definitive clinical validation remain incomplete [10,103,104]. A comparison of extracellular vesicles and exosomes is presented in Table 3 [50,80,82,85,87,89,96,97,101-116].

**Table 3:** Comparison of extracellular vesicles (EVs) and exosomes.

	EVs	Exosomes
Definition	Broad membrane-bound vesicle class released by cells, including exosomes and microvesicles [80,86].	Small EV subtype, usually 30-150 nm, generated through the endosomal multivesicular body pathway [50,104].
Preparations	Derived from cell culture with variable isolation methods and substantial heterogeneity [80,82].	Require enriched small-EV isolation and careful quality control; can also be engineered [102-104].
Mechanisms	Modulate inflammation, angiogenesis, apoptosis, matrix remodeling, and tissue repair [87,89,96].	Share similar functions but may offer more defined signaling and better platform standardization [106-108].
Indications	Broad musculoskeletal applications, including osteoarthritis, tendon and ligament healing, disc degeneration, and bone remodeling [87,89,96].	Targeted osteoarthritis, tendon and ligament repair, bone and cartilage regeneration, and other cell-free uses [110-115].
Advantages	Flexible umbrella platform that captures multiple vesicle types [80,85].	More homogeneous, engineerable, and scalable than mixed EV preparations [106,115].
Limitations	Heterogeneity and inconsistent nomenclature limit comparability [80,82].	More demanding quality control and manufacturing; standardization remains difficult [102,116].
Safety	Non-replicating and cell-free, but still investigational [87,97].	Early safety signals are encouraging, but there is still no FDA approval [105,113].
Evidence	Strong mechanistic interest, but still pre-standardization [80,87,101].	Validation is still early [10,113,116].

Gene Therapy and Gene-Edited Cell Therapies

Gene therapy refers to the delivery of nucleic acids (through viral or non-viral vectors, either in vivo or ex vivo) to alter gene expression in target cells. This approach includes gene addition, silencing, or editing to regulate growth factors, cytokines, or matrix proteins in musculoskeletal tissues. Gene therapies primarily target cell behavior at the molecular level [105].

Articular cartilage injuries associated with subsequent inflammation have limited potential for self-repair, as proinflammatory mediators like interleukin-1β (IL-1β) and TNF-α drive progressive cartilage degradation [100]. Recent research demonstrates that clustered regularly interspaced short palindromic repeats (CRISPR-Cas9) technology enables precise genetic modification. These advances allow for specific genetic edits that inhibit key nuclear factor-kappa B (NF-κB) pathway activators, yielding inflammation-resistant chondrocytes with superior regenerative potential and the ability to restore functional cartilage when combined with tissue engineering, including cells, scaffolds, and signals [106,107]. TGF-β-activated kinase 1 knockout (TAK1-KO) chondrocytes exhibit markedly reduced NF-κB activity and diminished production of inflammatory cytokines (e.g., IL-1, IL-6, and TNF-α) and catabolic mediators (e.g., MMP-13 and ADAMTS-5) after stimulation with IL-1, TNFα, or lipopolysaccharide (LPS). This approach supports the development of off-the-shelf, gene-edited, inflammation-resistant cartilage grafts, such as those utilizing allogeneic polydactyly chondrocytes embedded within a hyaluronic acid (HA) hydrogel to treat cartilage defects [106].

Gene-based approaches are entering early clinical application in musculoskeletal regenerative medicine, especially for cartilage and joint disorders [105,108]. Gene-edited chondrocytes and MSCs can be engineered to promote anabolic repair, resist inflammatory degradation, or produce therapeutic factors, often paired with biomaterial scaffolds and structured rehabilitation to maximize mechanical and biological outcomes [105,107,108]. Several early-phase clinical trials of these gene-modified cell products are underway, with some receiving expedited regulatory designations such as the FDA’s Regenerative Medicine Advanced Therapy (RMAT) status. This status highlights their promise for ongoing, localized protein delivery that could alter the trajectory of osteoarthritis and related chronic pain conditions rather than offering only symptomatic relief [108,109].

Progress in gene therapy and tissue engineering has accelerated innovation in tissue and organ regeneration. However, important technical, manufacturing, and translational barriers remain, and safety concerns such as immunogenicity, genotoxicity, and long-term effects are not yet fully resolved. Although some gene therapies are FDA-approved in other disease areas, musculoskeletal applications remain investigational [107,108].

Tissue Engineering and Biomaterial Scaffolds

Tissue engineering is defined as the creation or regeneration of tissue using a combination of reparative cells, scaffolds, and signaling factors to restore damaged structures and function (e.g., engineered cartilage or bone constructs for osteoarthritis) [110]. This discipline primarily targets rebuilding the tissue as a whole, combining cells, scaffolds, and factors into an implantable construct or in situ regenerative strategy. Accordingly, tissue engineering integrates three components: cells, growth factors, and three-dimensional scaffolds providing mechanical support and guiding tissue organization [111,112]. Immuno-engineering is a branch of regenerative medicine that focuses on the immune system and aims to modify the cellular response to facilitate tissue reconstruction [22,113].

Biomaterial scaffolds are defined as natural, synthetic, or hybrid 3D materials implanted to guide cell attachment, migration, and ECM formation; they may be acellular or cell-laden and can also serve as carriers for drugs or genes [114,115]. Biomaterial scaffolds serve as the structural component of many tissue engineering approaches and can be used with or without gene therapy (e.g., gene-activated matrices that release DNA/small interfering RNA in cartilage or bone defects) [116,117].

Scaffold materials for musculoskeletal regeneration rely on both natural and synthetic materials. Natural matrices such as hyaluronic acid, collagen, fibrin, and gelatin closely resemble the native extracellular matrix and are highly biocompatible. In contrast, synthetic polymers such as poly(lactic-co-glycolic acid) (PLGA), poly-L-lactic acid (PLLA), and polycaprolactone, which allow precise control over mechanical properties and controlled degradation kinetics. Hybrid scaffolds are particularly attractive because they combine both classes, merging biological advantages of natural materials with the structural reliability of synthetics to optimize tissue integration and regenerative outcomes [112,117]. Integration of gene therapy and gene editing tools, including vectors encoding growth factors, anti-inflammatory cytokines, matrix metalloproteinase (MMP) inhibitors, and CRISPR-Cas9, with scaffolds and cells can further improve mechanical strength, bio-functionality, and controlled degradation profiles [109,117,118].

Further directions involve utilizing pre-banked allogeneic cells and expanding these approaches from the knee joints to other anatomical sites, including the temporomandibular joint (TMJ), facet joints, and hips. This will require customizing scaffolds to match each joint's unique mechanics and developing immune-engineered therapies to tackle advanced OA and RA via immune engineering, and the need for academics to master the “translational vector” (regulation, manufacturing, intellectual property (IP), funding) to bring new therapies to patients [111,119].​ Ultimately, translating these innovations to the clinical setting will require academics to master the “translational vector,” which demands smart regulatory planning, scalable production, solid IP protection, and reliable funding streams (Table 4) [119,120].

**Table 4:** Comparison of gene therapy, tissue engineering, and biomaterial scaffolds. CRISPR: clustered regularly interspaced short palindromic repeats; PLGA: poly(lactic-co-glycolic acid); PCL: polycaprolactone.

	Gene therapy	Tissue engineering	Biomaterial scaffolds
Definition	Delivery of nucleic acids or editing systems to alter gene expression in tissues [117-119].	Uses cells, scaffolds, and signaling factors to restore tissue form and function [121-124].	3D materials that support cell attachment, matrix formation, and controlled bioactive delivery [109,111,114-116].
Preparations (sources)	Viral/non-viral vectors, CRISPR-based systems, ex vivo modified cells [117,120].	Autologous or allogeneic cells, bioreactors, and 3D bioprinting [123-125].	Collagen, hyaluronic acid, fibrin, PLGA, PCL, hydrogels, and hybrid matrices [114-116,126-128].
Mechanisms	Produces sustained local protein expression or suppression to change tissue biology [117,118,120].	Creates engineered constructs for mechanical support and biologic repair [122-124].	Adds structure and controlled release capacity to support integration and regeneration [109,114-116,124].
Indications (clinical use)	Cartilage injury, osteoarthritis, and other joint disorders [117,120,121].	Osteochondral defects and cartilage repair [122,123,125].	Cartilage, bone, and related musculoskeletal defects often serve as an enabling platform [109,111,114-116].
Advantages	Precise molecular targeting and potential for durable effects [105,108].	Addresses both biologic and structural deficits [123,124].	Flexible and can be tuned for mechanics, degradation, and local delivery [109,114-116,129,130].
Limitations	Off-target effects, immunogenicity, cost, and regulatory complexity [119,120].	Cell sourcing, integration, durability, and scale-up remain difficult [123,131].	Matching host mechanics, controlling degradation, and reproducible manufacturing remain challenges [109,114-116,128].
Safety	Mostly investigational in musculoskeletal care [119,121].	Early clinical translation, but not routine practice [123,131].	Strong research momentum, but use remains heterogeneous [109,111,114-116].
Evidence	High scientific promise, but early clinical maturity [117,120,121].	Important future platform, when combined with cells or biologics [122,123,131].	Best viewed as an enabling technology that is generally combined with cells, growth factors, genes, or other biologic signals rather than used as a stand-alone therapy [109,111,114,126].

Comparative effectiveness

Although regenerative therapies have shown encouraging results, their comparative effectiveness remains heterogeneous across different conditions. Among biologic modalities, PRP currently possesses the most consistent clinical evidence, particularly in the management of osteoarthritis and tendinopathy. Conversely, MSC-based therapies show great potential for genuine tissue regeneration but remain constrained by high costs, differences in preparation protocols, and ongoing regulatory hurdles [121-123].

Cell-free therapies, including extracellular vesicles and exosomes, are attracting significant attention due to their potential advantages in safety, storage, and scalability. However, robust clinical evidence supporting their routine clinical use is still lacking [121,124].

Overall, no single regenerative modality has proven consistently superior across all musculoskeletal conditions. Accumulating evidence supports a multimodal and individualized approach, where treatment selection is guided by the specific tissue involved, the stage of disease, and patient-specific factors (Tables 5, 6) [123-125].

**Table 5:** Comparative summary of regenerative and adjunctive therapies for musculoskeletal pain.

Therapy	Mechanism of action	Evidence	Indications	Advantages	Limitations	Clinical role
Platelet-rich plasma (PRP)	Releases growth factors that promote repair and modulate inflammation [109-111].	Moderate-high	Osteoarthritis, tendinopathy, and mild cartilage injury [109-111].	Autologous, widely available, and generally safe [109-111].	Preparation variability and limited standardization [109-111].	First-line biologic option.
Platelet-poor plasma (PPP)	Contains anti-inflammatory plasma proteins with fewer growth factors [109].	Low	Adjunct to PRP protocols and selected inflammatory conditions [109].	Low cost, simple preparation, and favorable safety [109].	Limited standalone evidence and unclear comparative efficacy [109].	Adjunct or investigational.
Mesenchymal stem cells (MSCs)	Combine paracrine signaling with differentiation to support tissue repair [109-111].	Low-moderate	Osteoarthritis, cartilage defects, and early degeneration [109-111].	Potential for structural repair and longer-term biologic effect [109-111].	Cost, heterogeneity, and regulatory barriers [109-111].	Second-line or specialized use.
Exosomes/extracellular vesicles	Cell-free signaling supports immunomodulation and regeneration [110-111].	Low	Experimental use in osteoarthritis and cartilage injury [110,111].	Cell-free, scalable, and low immunogenicity [110,111].	Limited high-quality clinical trials [110,111].	Investigational or future therapy.
Gene therapy	Gene transfer enables sustained production of therapeutic proteins [113].	Low	Experimental use in cartilage disorders and osteoarthritis [113].	Targeted, potentially long-lasting biologic effect [113].	Safety concerns, cost, and regulatory complexity [113].	Emerging or experimental.

**Table 6:** Overview of regenerative medicine modalities. Overview of regenerative medicine modalities used in musculoskeletal pain management. Blood-derived biologics, cell-based therapies, cell-free therapies, gene therapy, and tissue engineering are illustrated together with their sources, principal mechanisms of action, and major clinical applications. MSCs: mesenchymal stromal cells; PRP: platelet-rich plasma; PPP: platelet-poor plasma; PDGF: platelet-derived growth factor; TGF-β: transforming growth factor-beta; OA: osteoarthritis; LBP: low back pain.

	PRP	PPP	MSCs	Gene therapy	Tissue engineering
Source	Patient blood	Fraction of PRP process	Bone marrow, adipose tissue, Wharton’s Jelly	Engineered cells/vectors	Biomaterial scaffolds + patient cells
Mechanism of action	Concentrated growth factors (PDGF, TGF-β) stimulate angiogenesis and cell migration	Reduces inflammation, modulates scarring, muscle differentiation	Immuno-modulation differentiation into repair tissues (bone/cartilage)	Delivers therapeutic genes to express growth factors or block inflammation	Provides structural support, guides host cell regeneration
Indication and uses	OA, tendinopathy, LBP	Acute muscle injury, athletic return-to-play	Knee OA, disc degeneration, rheumatoid arthritis	Severe cartilage defects, genetic joint disorders	Osteochondral defects, complex bone voids

Future directions

Emerging technologies are transforming regenerative strategies for musculoskeletal pain by integrating biologically active therapies, smart biomaterials, and digital innovations to improve the precision and durability of interventions [121,126,127]. Artificial intelligence (AI) and machine learning models are increasingly used to predict disease progression, stratify phenotypes of osteoarthritis and chronic low back pain, and identify responders most likely to benefit from PRP or MSCs [128,129]. These innovations support a move toward precision regenerative pain medicine. AI-assisted image analysis is being explored to improve anatomical localization and procedural targeting during musculoskeletal interventions. Machine-learning approaches are also being investigated for scaffold design and optimization in bone and cartilage tissue engineering. AI-based clinical decision support may assist individualized rehabilitation planning, although clinical decision support remains limited [127,130-137].

Regarding biological therapeutics, new-generation cell-free products, such as MSC-derived extracellular vesicles and exosomes, are being engineered as standardized, ready-to-use nano-carrier delivery systems. These approaches aim to replicate or exceed the paracrine benefits of MSCs while minimizing immunogenicity and tumorigenicity [132,133]. Parallel advances in gene therapy and gene-edited cell products, including CRISPR-modified chondrocytes or MSCs resistant to inflammatory degradation, as well as vectors encoding IL-1 antagonists or anabolic factors, are entering early-phase clinical testing for cartilage and joint disease. The overarching objective is to achieve sustained in situ protein production and durable modification of osteoarthritis trajectories rather than transient symptom relief [133,134].

Advances in tissue engineering and three-dimensional (3D) bio-printing enable the fabrication of osteochondral and ligament-like constructs that integrate cells, bioactive factors, and biomaterial scaffolds tailored to joint-specific mechanical demands. Hybrid scaffold systems that combine natural matrices (such as collagen or hyaluronic acid) with synthetic polymers and gene-activated components are being optimized to improve tissue integration, regulate degradation kinetics, and deliver genes or EVs locally in a spatiotemporally controlled manner [135,136].

First, resolving clinical heterogeneity requires immediate consensus on preparation protocols for blood- and cell-derived products. Establishing uniform metrics for cellular dosing, purity, preparation methods, and localized delivery parameters is essential to improve reproducibility and enable meaningful comparison across studies [137-139]. Second, long-term, adequately powered RCTs with harmonized outcome measures (pain, function, structure, and biomarkers) are needed to clarify comparative effectiveness versus conventional care and between regenerative modalities in specific phenotypes of OA, tendinopathy, axial pain, and muscle injuries. Third, precision medicine approaches integrating clinical features, imaging, molecular profiling, and AI prediction could yield individualized regenerative treatment algorithms, selecting between cell-based, cell-free, scaffold, and mechano-based strategies according to patient- and tissue-specific characteristics [137-140]. Finally, translational expertise in regulatory science, manufacturing, and health economics will be essential to move gene therapies, EV/exosome platforms, and advanced tissue-engineered constructs from experimental use into accessible, ethically sound clinical practice for chronic musculoskeletal pain. Furthermore, although some regenerative modalities are increasingly used in clinical practice, many advanced biologics and gene-based approaches remain investigational according to regulatory agencies, underscoring the need for larger, long-term studies, standardized manufacturing processes, and clearer regulatory pathways before broader routine implementation can be recommended [138,139,141].

Limitations of this review

This review has several important limitations that should be acknowledged when interpreting its findings.

First, while the literature search covered multiple databases over a 10-year window, the synthesis remained narrative rather than fully systematic, and a formal meta-analysis was not performed; therefore, effect size estimates and between-study variability could not be quantitatively pooled.

Second, considerable heterogeneity exists across the included studies with respect to patient demographics, anatomical sites, disease severity, preparation protocols, injection techniques, comparators, and follow-up duration, which limits direct comparability and may have introduced clinical and methodological heterogeneity.

Third, as with many emerging therapeutic fields, publication bias is possible, as positive or novel regenerative interventions (such as MSC products and exosome protocols) are more prone to publication than negative or neutral studies.

Fourth, musculoskeletal pain is multifactorial and often accompanied by psychosocial, biomechanical, and comorbid conditions; however, most studies focused on structural or biological endpoints and did not consistently account for psychological distress or central sensitization, which may confound observed treatment effects.

Fifth, safety data were frequently limited to short- or intermediate-term follow-up (often six to 24 months). Consequently, rare adverse events, long-term durability of benefit, and potential risks, such as heterotopic ossification, accelerated degeneration, or tumorigenicity, may be underreported for some advanced biologics.

Finally, this review was restricted to adult populations with musculoskeletal pain and excluded postoperative, cancer, and neuropathic pain, as well as animal and preclinical studies, which narrows generalizability and omits mechanistic insights from basic science work that could inform clinical translation. Consequently, the practical feasibility and economic impact of the broad adoption of some regenerative technologies remain incompletely addressed.

## Conclusions

Regenerative medicine is reshaping the conceptual landscape of musculoskeletal pain management by shifting attention from transient symptom suppression toward biologically informed tissue modulation and repair. Current evidence suggests that a broad range of modalities, including PRP and PPP, MSCs and their derivatives, exosomes and EVs, tissue engineering, biomaterial scaffolds, and gene-based interventions, may improve pain and function and have shown promising results regarding pain control and function and, in selected settings, may also support structural recovery. However, the magnitude and durability of these benefits remain inconsistent, with substantial variability in protocols, methodological quality, and limited long-term follow-up. For this reason, many regenerative interventions are best viewed as adjuncts to, rather than replacements for, comprehensive multimodal care. No single therapy appears universally superior, and treatment choice should depend on the underlying pathology, disease stage, and patient factors.

Future progress will depend on standardized protocols, harmonized reporting of biologic products, validated outcome measures, long-term safety data, and robust cost-effectiveness studies. Advances in precision medicine, AI-assisted stratification, cell-free biologics, bioengineering scaffolds, and gene-based technologies may further expand therapeutic options. Their meaningful translation into routine practice, however, will require close collaboration among clinicians, researchers, bioengineers, industry, and regulators to ensure that innovation is matched by rigorous evidence, reproducible manufacturing, and patient-centered benefits.
